# Disturbances of Dental Development in Cleidocranial Dysplasia

**DOI:** 10.1097/SCS.0000000000011287

**Published:** 2025-03-27

**Authors:** Vincent R. The, Brunilda Dhamo, Eppo B. Wolvius

**Affiliations:** * Department of Oral & Maxillofacial Surgery, Special Dental Care and Orthodontics, Erasmus University Medical Centre; † The Generation R Study Group, Erasmus University Medical Centre, Rotterdam, The Netherlands

**Keywords:** Cleidocranial dysplasia, dental age, dental development, supernumerary teeth

## Abstract

**Objective:**

This study aimed to investigate the delay in dental development in patients with cleidocranial dysplasia (CCD) applying a longitudinal approach of maturational stages of teeth. A secondary objective was to examine whether an increasing number of supernumerary teeth influences dental development.

**Methods:**

A total of 38 patients were included, consisting of 13 patients with CCD and 25 without CCD, selected from the Generation R Study. Both groups were matched by age and number of supernumerary teeth.

Dental development was assessed using the Demirjian method. Linear mixed models were used to study the longitudinal aspects of dental development delay between the 2 timepoints. Subgroup analysis was performed using 3 linear regression models to study the extent of dental development delay in CCD patients compared with the control group and to evaluate the influence of an increasing number of supernumerary teeth on developmental delay.

**Results:**

No significant decelerations in dental development between late childhood and early adolescence were found in both groups. However, the CCD group showed a delay in dental development of 3.3 years [β −0.8; 95% CI (−4.3,−2.2)] in comparison to the control group when confounded for sex and supernumerary teeth. The number of supernumery teeth proved not to be a significant determinant as there was a *P*-value of 0.22.

**Conclusions:**

The authors' findings indicate that dental development in CCD patients is delayed by ~3.3 years, which stays stable from adolescence. An increasing number of supernumerary teeth did not contribute to further delays in dental development.

Cleidocranial dysplasia (CCD; OMIM 119600) is an autosomal dominant inheritance disorder with an estimated prevalence of one per million newborns that does not differ by ethnic background or by sex.^1^ CCD is caused by mutations in the runt-related transcription factor-2 (RUNX2) gene, which plays an important role not only in osteoblast differentiation and in osteoclastogenesis in the dental follicle, the periodontal ligament, and presumably in the craniofacial bones, but also in resorption of the dental lamina when the formation of the replacement teeth is initiated.^2,3^ As a result, CCD is associated with a variety of skeletal, craniofacial, and dental abnormalities. Skeletal manifestations include hypoplastic or aplastic clavicles, patent sutures and fontanels, short stature, and Wormian bones.^4^ The disturbed craniofacial growth of CCD is shown in the underdevelopment of the maxilla and the delayed fusion of the mandibular symphysis.^5,6^ Dental abnormalities, such as retention of deciduous teeth, impacted supernumerary teeth, and delayed eruption of permanent teeth are commonly manifested in ~94% of CCD patients.^7–9^ Retained teeth, including supernumerary germs, can lead to the formation of dentigerous cysts, necessitating continuous monitoring of the dentition in CCD patients.

Supernumerary teeth are a known possible cause of delayed eruption due to physical obstruction of the eruption pathway on adjacent teeth.^10,11^ However, the impact of the supernumerary tooth on the formation and maturation of adjacent teeth is less known. A retrospective study performed by Kan et al^12^ found no significant difference in dental development between children with non-sydromic hyperdontia and healthy controls.

Most of the studies looking into the relationship between CCD and delay in dental development used the time of eruption of the teeth to estimate the dental delay.^3,13–15^ To our knowledge, only 3 studies have investigated dental maturation to assess delay in dental development in CCD patients. Jensen and Kreiborg were the first to study dental development in CCD. They conducted a longitudinal study in 1990 and found a delay in dental development of 1 to 4 years.^16^ However, this study lacked the use of a control group. A cross-sectional study by Seow et al^17^ in 1995 reported a delay of 3.1 years compared with a healthy control group. Shaikh and Shusterman also performed a cross-sectional study in 1998 and concluded that CCD patients have a delay of 2.1 years.^18^ If supernumerary teeth were present in these CCD patients, there was an additional delay of 1.5 years, although not statistically significant. None of these studies, however, consisted of a longitudinal setup with a control group.

The primary objective of this retrospective study was to investigate the delay in dental development in CCD patients applying a longitudinal approach of maturational stages of teeth. The secondary objective was to study whether the increase in a number of supernumerary teeth influences dental development in individuals with and without CCD.

## MATERIALS AND METHODS

Ethical approval from the Medical Ethical Committee of the Erasmus Medical Centre, Rotterdam, the Netherlands was acquired before any data collection. The study was conducted in accordance with the Declaration of Helsinki (October 2013) and in full compliance with the regulations of Medical Research Ethics Committee (METC) of the Erasmus Medical Centre in Rotterdam, the Netherlands (MEC-2021-0073).

### Study Population

Participants included in the study were: (1) patients diagnosed with CCD who have been examined and treated at the Department of Oral & Maxillofacial Surgery of Erasmus Medical Centre in Rotterdam, the Netherlands, or (2) participants of the Generation R Study with at least one supernumerary tooth germ observed in the dental panoramic radiograph (DPR) that has been taken as part of the Generation R Study protocol. Supernumerary teeth needed to be present in both groups to study the influence of an increasing number of supernumerary teeth on dental development. Inclusion criteria for CCD patients were a diagnosis of CCD by clinical and radiological examination and at least 2 available DPRs in the patient’s record. Exclusion criteria included individuals over 18 years old with fully developed dentition and those with no contact records or available DPRs.

Records of 23 CCD patients were available. As 3 of the patients had no available DPR and 7 of the patients were already fully matured in dental development at the time of the first DPR, only 13 patients fulfilled the eligibility criteria for inclusion. All included CCD patients had 2 DPRs available.

Participants from the control group were selected from the Generation R Study. The Generation R Study is a multi-ethnic population-based prospective cohort study from fetal life onwards, which was initiated to identify early environmental and genetic determinants of growth, development, and health. All children were born between April 2002 and January 2006. Inclusion criteria for the control group were healthy individuals without CCD or any other syndrome, the presence of one or more supernumerary teeth, and at least one available DPR in the participant’s record. Exclusion criteria were the same as those for the CCD group.

Of 9285 participants with 1 or 2 available DPRs, 42 participants had one or more supernumerary teeth. Out of these 42 healthy individuals, 17 had a completed development of the dentition. The remaining 25 participants were used as the control group. Out of these 25 individuals, 17 had only 1 DPR available and 8 participants had 2 DPRs available.

### Assessment of Dental Development

Dental development was defined from each available DPR using the Demirjian method.^19^ The development stage (A, B, C, D, E, F, G, or H) of each tooth in all quadrants was determined by one examiner (BD).^20,21^ Dental age was calculated for each patient referring to developmental stages of teeth in the lower left quadrant. If teeth were missing in the left mandibular quadrant, the developmental stages of the missing teeth were ascertained from a combined method. A corresponding tooth was used from the right mandibular side or from the corresponding maxillary tooth when the tooth was missing in both sides of the mandible. In case no corresponding tooth was present, regression equations developed by Nystrom et al^22^ were applied. These equations take into account the development of the remaining teeth in the lower left quadrant, age and sex of the patient to calculate dental age. Obtained stages of development were used to calculate the dental maturity score by summing up the weighted scores given to every tooth of the lower left quadrant.^23^ Then, the Dutch dental age standard for boys and girls was used to convert the dental maturity score into dental age.^23^ Finally, the difference between the dental age and the chronological age was calculated [dental age (DA) − chronological age (CA)] and used as the main outcome.

### Statistical Analysis

All statistical analyses were performed using the statistical software Statistical Package for Social Sciences version 24.0 (SPSS Inc.).

The intraclass correlation coefficient was calculated to determine the agreement of the stages of development (A to H) for each of the 7 left mandibular teeth between 2 independent examiners (V.T. and B.D.). A subsample of 12 DPRs was used, which corresponds to 20 percent of the total number of DPRs in the study population. Independent *t* test were used to test the differences in patient characteristics between the CCD group and control group. To analyze the effect of the CCD condition on dental development, linear mixed models were used in a longitudinal setup. Linear regression models were used for cross-sectional analysis to assess the delay in dental development in CCD and the role of supernumerary teeth. We built 3 models, each adjusting for different confounders. Model 1 was a crude analysis without any confounders. Model 2 was a confounding model, adjusted for sex. Model 3 was adjusted for sex and number of supernumerary teeth.

Before performing the study, we calculated the sample size (N) needed for our study. For this study we accepted a level of significance *P*<0.05.

## RESULTS

### General Characteristics of the Study Participants

The patient characteristics are presented in (Supplemental Table 1, Supplemental Digital Content 1, http://links.lww.com/SCS/H591). Among the 13 patients with CCD, 5 were male and 8 patients were female. The CCD patients had a mean age of 11.1 years (±2.8) at the first DPR and 13.5 years (±2.7) at the second DPR. The dental age at the first and second timepoints was 8.6 years (±2.0) and 11.5 (±2.8), respectively. The difference in dental age and chronological age was −2.5 years (±2.1) for the first timepoint and −2.0 (±1.4) for the second timepoint. Out of the 13 patients, there was only one patient with dental fillings at the first timepoint. At the second timepoint there was one additional filling in another patient.

In the control group, there were 18 males and 7 females, with a mean age of 10.3 years (±1.4) at the first timepoint and 13.4 years (±0.1) at the second timepoint. The corresponding dental ages were 10.7 years (±1.2), and 14.1 years (±2.2). The difference between dental age and chronological age was 0.4 years (±0.9) at the first timepoint and 0.6 years (±2.2) at the second timepoint. Among the 25 control participants, 17 had only 1 DPR available, whereas 8 had 2 DPRs available.

The sample size calculation resulted in a required number of 18 participants.

### Inter-examiner Agreement for the Study Population

The inter-examiner agreement resulted excellent (ICC>0.90) for all teeth except the first molars for which a good agreement (ICC=0.90) was achieved.

### Longitudinal Aspect of Delay in Dental Development

**FIGURE 1:**
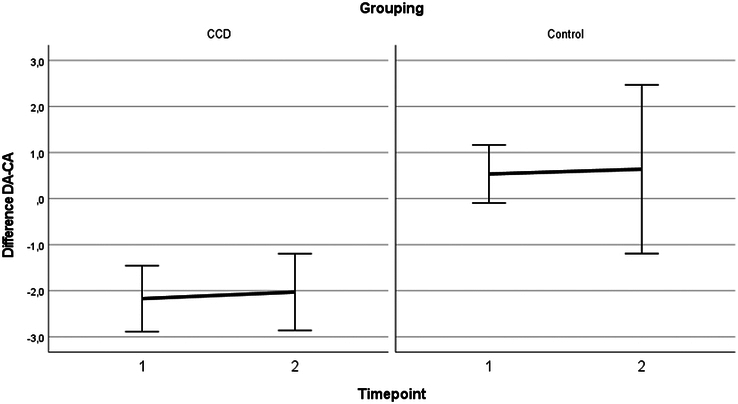
Difference between dental age and chronological age with the 95% CI at Timepoint 1 and 2 for both the CCD group and control group. This illustrates a consistent delay in dental development in the CCD patients.

Estimated fixed effects of the linear mixed model analysis can be found in (Supplemental Table 2, Supplemental Digital Content 1, http://links.lww.com/SCS/H591). These show no statistically significant decelerations in dental development in both the CCD and the control group. Figure 1 displays the dental development. In the CCD group, the delay was consistent at both timepoints, with a delay of −2.2 years [95% CI (−2.9,−1.5)] at age 11.1 and a delay of −2.0 years [95% CI (−2.9,-1.2)] at age 13.5. In contrast, the control group showed a slight acceleration in dental development, with a delay of 0.6 years (95% CI: −1.2, 2.5) at age 9.7, and 0.5 years (95% CI: −0.1, 1.2) at age 13.4.

### Delay in Dental Development and Effect of Supernumerary Teeth

Results from the linear regression analysis are presented in (Supplemental Table 3, Supplemental Digital Content 1, http://links.lww.com/SCS/H591). In model 1, CCD patients showed a significant delay of 2.6 years [β −0.8; 95% CI (−3.3,−1.9)] in comparison to the control group. In model 2 a significant delay of 2.8 years [β −0.9; 95% CI (−3.5,−2.1)] for the CCD group was shown. Sex proved not to be a statistically significant determinant. In the fully adjusted model 3, which confounded for both sex and number of supernumerary teeth, the delay increased to 3.3 years (β=−1.0, 95% CI: −4.1, −2.2) A significant effect in sex as a determinant was found, where males have a higher delay of 0.7 years [β −0.2; 95% CI (−1.4, −0.03)]. The number of supernumerary teeth proved not to be a significant determinant as there was a *P*-value of 0.22. The unstandardized beta of a number of supernumerary teeth was 0.2.

## DISCUSSION

The findings of our study suggest that CCD patients manifest a delay in dental development of ~3.3 years. The delay in dental development is in line with studies conducted in the past, indicating delays ranging from 1 to 4 years.^16–18^

Notably, our data suggest that this delay remains stable from late childhood (around age 11) to early adolescence (around age 13), with no significant acceleration or deceleration in development during this period. We hypothesize that the delay in development remains to be stable, even after early adolescence, leading to CCD patients having a fully matured dentition about 3 years later than healthy individuals.

According to Jensen and Kreiborg, the development of primary dentition in CCD patients is typically normal.^16^ Their study reported no delay in dental development at ages 5 and 6, but identified an ~1-year delay in the 7 to 9 age group, with a progressively greater delay observed in older age groups. However, Jensen and Kreiborg did not use standardized timepoints for follow-up, so the number of evaluations and the age at which they occurred varied. Their data suggest that the initiation of secondary tooth development occurs normally, but a delay in dental development likely begins after the age of 6. This delay would then increase until around ages 10 to 11, after which it seems to stabilize until full dental maturation is achieved.

The lack of further progression in the delay after age 11 may be explained by the onset of the growth spurt. A recent meta-analysis showed a high correlation between dental development and skeletal age.^24^ Multiple studies showed that the beginning of the growth spurt can be indicated by canine stage F.^25,26^ As most of our included CCD patients (62%) were at this stage at the first timepoint, or one stage lower (31%), it is possible that puberty and the start of the growth spurt are time phases where dental development in CCD starts to catch up. The role of the growth spurt can be further substantiated by our finding that the delay in dental development in females was less than in males, as females tend to start puberty at an earlier age. Hormonal changes could be the bases for the acceleration in dental development. Further research into this topic would be needed, however, to test this theory.

The delay in dental development observed in CCD patients is believed to be caused by mutations in the RUNX2 gene. RUNX2 influences various downstream targets in different regions of the jaw and plays multiple roles in dental development. It is important for both crown formation and root development. In vivo studies in mice have demonstrated that RUNX2 is essential for tooth development from the cap stage to the bell stage.^27^ These stages are crucial for the formation of the enamel knot and the enamel organ epithelium, which are necessary for tooth enamel development. In addition, RUNX2 is vital for odontoblastic differentiation, mediated through one of its downstream targets, NOTUM.^28^ RUNX2 is also expressed in GLI1+ root progenitor cells, and its loss in these cells, as well as in their progeny, leads to defects in root development. Therefore, RUNX2 plays a critical role in both tooth development and in regulating the differentiation of root progenitor cells.

The clinical relevance of our study lies in the observed stability of dental development delay from around the age of 11 years. For CCD patients aged 11 or older, dental development delay can be reliably calculated, allowing for a more personalized estimation of when full dental maturation is likely to occur. This information can be particularly useful in treatment planning. Effective management of CCD often requires interdisciplinary collaboration among orthodontists, maxillofacial surgeons, and general dentists.^2,29^ Commonly required treatments for cleidocranial dysplasia include extractions, and surgical exposure of retained elements with orthodontically aided eruption. The latter procedure is ideally carried out when the roots of the impacted teeth have developed to about two-thirds of their full length.^2^ Depending on the tooth, this is typically expected to occur between the ages of 8 and 14 in a normal population. In CCD, however, according to our findings, this will generally occur up to 3 years later. This may help avoid the premature taking of radiological images in anticipation of such treatments for patients diagnosed with CCD. However, if the optimal timing for orthodontic alignment is neglected, this can result in tooth loss, malocclusion disorders, or more extensive treatments such as dental implants. Therefore, early diagnosis and treatment planning are one of the key factors in managing CCD.^2^ Ideally, CCD should be diagnosed before the age of 11, by which time we believe delayed dental development is already visible on radiological imaging. This allows for the development of a treatment plan that includes surgical exposure and orthodontically assisted eruption, taking into account the delayed dental development. Another complicating factor in treatment planning is that, along with delayed dental development, there may also be delayed eruption, likely due to abnormal bone resorption associated with CCD.^16^ As a result, it will be essential to develop an individualized treatment plan for each CCD patient.

Clinicians can consider the use of the calcification stages as a screening method for CCD. Naturally, a delayed time of eruption is an easier sign to notice than a delayed calcification stage in the diagnosis of CCD. However, radiographs are taken quite regularly by orthodontists, general dentists or other oral health care workers, and screening for any delayed calcification stages can help in early diagnosing CCD. The time of eruption of the secondary teeth in CCD namely, is widely variable as it is dependent on multiple factors such as diminished bone resorption, diminished resorption of the roots of the primary teeth, and physical barriers such as supernumerary teeth.^3^

In addition, an increasing number of supernumerary teeth does not seem to have an effect on the delay in dental development, as the number of supernumerary teeth did not prove to be a significant determinant. To our knowledge, this is the first study to look at the effect of the number of supernumerary teeth instead of looking at the effect of the presence of supernumerary teeth alone.

Shaikh et al^18^ studied the effect of supernumerary teeth on delay in dental development in CCD patients. They found that if CCD patients had supernumerary teeth there was an additional delay of 1.5 years in comparison to CCD patients without supernumerary teeth. These findings, although, were based on small patient numbers and were not statistically significant. A study from Mallineni et al^30^ found that not only the number of supernumerary teeth might be associated with developmental delay but also the localization of these supernumerary teeth. Mallineni found that there was no significant delay in dental development in children with supernumerary teeth in comparison to a healthy control group, but when dividing the children with supernumerary teeth in a group with unilateral supernumerary teeth and a group with bilateral supernumerary teeth, there was a significant delay in boys with bilateral supernumerary teeth.^30^

Although the specific cause of delayed dental development in CCD remains unclear due to a lack of specific research, it is generally believed that the underlying pathogenesis is related to the general skeletal delay.^2,17^ Whether supernumerary teeth contribute to this delay by exerting local effects on adjacent teeth cannot be confirmed or refuted by this study, as we did not investigate causal relationships.

The CCD patients had a good condition of their dentition in terms of dental decay. The small number of CCD patients with fillings indicates that there is relatively good oral hygiene in CCD patients. A potential contributing factor is that CCD patients typically exhibit normal IQ development, unlike individuals with certain other craniofacial conditions. Consequently, they often maintain good oral hygiene at home, complemented by high-quality professional dental care.

Some strengths and limitations need to be recognized in this study. We studied dental development in patients with CCD in a longitudinal aspect together with a control group, which has not been previously reported.

The limitations are mainly related to the number of participants included. The inclusion of more CCD patients would have improved statistical power. Our calculated sample size was 18, whereas we were only able to include 13 CCD patients, due to the rarity of the condition. In addition, the control group would have benefited from a higher number of participants with at least 2 DPRs. Because of ethical considerations with respect to the “as low as reasonably achievable” (ALARA) principle a higher number of these participants were not available. To gain a more comprehensive understanding of the delay in dental development, future studies should include a larger cohort of CCD patients across a broader age range, ideally beginning at age 6, with regular radiographic follow-up. A prospective study with a multicenter setup may be needed to explore the delay in dental development in CCD further.

## CONCLUSIONS

According to our study, patients with CCD have a delay in dental development of ~3.3 years in comparison to a healthy control group with supernumerary teeth. The delay in dental development did not significantly increase, nor decrease from ages 11.1 to 13.5, from which it seems likely that the delay in dental development stays stable from the age of around 11 years old until full maturation. Furthermore, we found no significant increase in dental development delay with an increasing number of supernumerary teeth.

## Supplementary Material

SUPPLEMENTARY MATERIAL
