# Supplementary material for: Disturbances of Dental Development in Cleidocranial Dysplasia
Source: J Craniofac Surg. 2025 Mar 27;36(8):3070–4. doi: 10.1097/SCS.0000000000011287 (PMC12537041; doi:10.1097/SCS.0000000000011287)
Supplement: SUPPLEMENTARY MATERIAL [file scs-36-03070-s001.docx]

Supplemental table 1: Characteristics of patients included in the study (N=38). Statistical significance was defined as a p-value of <0.05. The difference between dental age and chronological age shows the delay in dental development in the Cleidocranial dysplasia group.

|  | **CCD (N=13)** | **Controls (N=25)** | **p-value *** |
| --- | --- | --- | --- |
| **Sex *(N,%)* Boys Girls** | 5 (38.5%) 8 (61.5%) | 18 (72%) 7 (28%) | <0.05 |
| **Age T1 *(years; mean, SD)*** | 11.1, 2,8 | 10.3, 1.4 | 0.12 |
| **Age T2 *(years; mean, SD)*** | 13.5, 2.7 | 13.4, 0.1 | 0.47 |
| **Timespan *(years; mean, SD)*** | 2.4, 0.6 | 3.7, 0.1 | <0.05 |
| **Dental age T1 *(years; mean, SD)*** | 8.6, 2.0 | 10.7, 1.2 | <0.05 |
| **Dental age T2 *(years; mean, SD)*** | 11.5, 2.8 | 14.1, 2.2 | <0.05 |
| **Difference dental age – chronological age T1 (mean, SD)** | -2.5 (2.1) | 0.4, 0.9 | <0.05 |
| **Difference dental age – chronological age T2** | -2.0, 1.4 | 0.6, 2.2 | <0.05 |
| **Number of supernumerary teeth** | 3.9, 2.2 | 1.1, 0.3 | <0.05 |
| **Available DPR’s** |  |  |  |
| **Only 1 available DPR** | 0 | 17 |  |
| **2 available DPRs** | 13 | 8 |  |

N = number of patients, SD = standard deviation.

*Differences were tested using independent t-test.

Supplemental table 2: Longitudinal aspects of delay in dental development. The effect estimate shows the acceleration in dental development in years between timepoint 1 and timepoint 2 for both the CCD and the control group. Statistical significance was defined as a p-value of <0.05.

| **Grouping** | **Effect estimate** | **95% CI** | **p-value** |
| --- | --- | --- | --- |
| **CCD** | 0.1 | -0.3, 0.6 | 0,49 |
| **Control** | 0.1 | -1.3, 1.5 | 0.87 |

CCD = Cleidocranial dysplasia, CI = confidence interval.

Supplemental table 3: Difference of dental age and chronological age between the CCD group and control group. The unstandardized beta shows the years of delay in dental development of the CCD group in comparison to the control group. In Model 2 and Model 3 it also shows the effect of sex and supernumerary teeth. Statistical significance was defined as a p-value of <0.05.

|  | **Model 1** | | | **Model 2** | | | **Model 3** | | |
| --- | --- | --- | --- | --- | --- | --- | --- | --- | --- |
|  | **B** | **95% CI** | **p-value** | **B** | **95% CI** | **p-value** | **B** | **95% CI** | **p-value** |
| **CCD** | -2.6 | -3.3, -1.9 | <0.05 | -2.8 | -3.5, -2.1 | <0.05 | -3.3 | -4.3, -2.2 | <0.05 |
| **Sex** |  |  |  | -0.6 | -1.3, 0.07 | 0.08 | -0.7 | -1.4, -0.03 | <0.05 |
| **Supernumerary teeth** |  |  |  |  |  |  | 0.2 | -0.1, 0.4 | 0.22 |

Model 1- no confounders; Model 2- sex; Model 3- number of supernumerary teeth and sex. CCD = Cleidocranial dysplasia, CI = confidence interval, B = unstandardized beta.
